# A High Coordination of Cross-Links Is Beneficial for the Strength of Cross-Linked Fibers

**DOI:** 10.3390/biomimetics4010012

**Published:** 2019-02-04

**Authors:** Huzaifa Shabbir, Christoph Dellago, Markus A. Hartmann

**Affiliations:** 1 Faculty of Physics, University of Vienna, Boltzmanngasse 5, 1090 Vienna, Austria; huzaifa.shabbir@univie.ac.at (H.S.); christoph.dellago@univie.ac.at (C.D.); 2 Ludwig Boltzmann Institute of Osteology at the Hanusch Hospital of WGKK and AUVA Trauma Centre Meidling, 1st Medical Department Hanusch Hospital, Heinrich Collin Strasse 30, 1140 Vienna, Austria

**Keywords:** fiber bundles, cross-link coordination, computational loading experiment, strength

## Abstract

The influence of the coordination of (reversible) cross-links on the mechanical properties of aligned fiber bundles is investigated. Two polymeric systems containing cross-links of different coordination (two- and three-fold coordination) but having the same binding energy are investigated. In particular, the response to loading of these systems is compared. Mechanical parameters (strength, stiffness and work-to-fracture) are obtained by computational loading tests. The influence of coordination is studied for simple test systems with pre-defined topologies that maximize strength as well as for more realistic fiber bundles containing nine chains. The results show that a higher coordination of cross-links has a beneficial effect on the strength and the stiffness of the systems, while the work-to-fracture was found larger for the system having a smaller coordination of cross-links. It can be concluded that controlling the coordination of cross-links is a versatile tool to specifically tailor the mechanical properties of polymeric structures.

## 1. Introduction

Structural properties of polymers are rather independent from the properties of their backbones. A common strategy to specifically tailor the mechanical performance of natural and synthetic polymers is to (reversibly) cross-link the polymer [1]. A “cross-link” is an additional bond connecting distant parts of one polymer or two distinct polymers. One prominent technological example is the vulcanization of rubber. In this process, natural rubber is permanently cross-linked with sulfur bridges, thereby dramatically enhancing its stiffness, strength and wear properties [2,3].

In biological materials, cross-links are often considerably weaker than the covalent backbone that holds the structure together. Consequently, upon loading, these bonds rupture before the covalent backbone fails. The rupture of cross-links unveils the segments of the structure that were previously shielded from loading, unraveling what is picturesquely called “the hidden length” [4]. Cross-link rupture and hidden length unraveling are highly efficient mechanisms to dissipate large amounts of energy and obtain enhanced mechanical properties [5,6]. Furthermore, these bonds are often reversible, meaning that cross-links may reform after the load is released. Consequently, these biological materials show self-healing properties even in the absence of living cells [7]. In the biological context, such cross-links are often referred to as “sacrificial bonds” [4] and can be found in numerous materials such as mussel byssus threads [8,9], bone [10,11,12], wood [13], spider silk [14,15], or caddisfly silk [16]. The nature of these bonds ranges from weak hydrogen bonds in spider silk [17,18] to relatively strong metal-coordination bonds in the mussel byssus that have binding energies approximately a quarter the strength of the backbone [19,20].

These special mechanical properties of cross-links have aroused a considerable amount of theoretical and modeling effort to describe, understand and predict their structural and mechanical effects. These studies include the investigation of stiff networks [21], the unbinding transition of semiflexible polymer bundles [22], understanding the role of nanoconfined hierarchical assemblies of weak hydrogen bonds in β-sheet crystals in silk [23], the influence of network connectivity and entanglement on network mechanics [24], as well as calculating the force–extension relation of cross-linked polymers [25,26]. Also, the effect of hidden length opening on the mechanical properties of biological materials was explicitly studied [27,28,29].

In all these aforementioned studies, a cross-link is defined as an additional bond between either two parts of the same polymer (intrachain cross-link) or between two different polymers (interchain cross-link). Microscopically, this means that cross-links are modeled to form between two monomers. While this situation holds true in many materials, there are also important other classes of cross-links that connect more than two monomers. Typical examples of such cross-links are metal-coordination complexes, like the Fe–3,4-dihydroxyphenylalanine (DOPA) or Zn–histidine complex found in the mussel byssus [7,8,30]. Such a coordination complex consists of one metal ion bound to several organic ligands [31,32,33,34]. These metal complexes form fast and reversibly, have a high strength and provide the material with remarkable mechanical properties including high toughness and stiffness, enormous extensibility up to 100%, self-healing and exceptional adherence in wet conditions [7,9].

There are only a few studies that explicitly investigate the influence of the coordination of cross-links on the mechanical behavior. One exception are references [35,36] where di- and trivalent cross-links in collagen are studied. In the present paper, we generalize a model presented in our previous work [37] to study the effect of cross-linking a single chain to the important case of aligned fiber bundles. We refer to the “coordination” of cross-links as the number of ligands bound to a metal ion in a coordination complex (i.e., the number of monomers involved in one cross-link). A two-fold coordinated cross-link is referred to as a “dimer” or “bis-complex”, whereas a “trimer” or “tris-complex” refers to a three-fold coordinated cross-link. The deformation behavior of aligned fiber bundles that are cross-linked by three-fold or two-fold coordinated cross-links is compared. The physical properties of the cross-links are modeled using a generic approach given by the framework provided by the reactive empirical bond-order (REBO) potential [38,39] that gives the possibility to control the coordination of cross-links.

The obtained results indicate that interchain cross-links play a crucial role in the mechanical behavior of aligned, interacting chain bundles. As previously shown, the presence of cross-links may deteriorate the strength of aligned fiber bundles compared to the non-cross-linked case [40]. This effect is further analyzed in the current study and the results show that a higher coordination of cross-links may partially restore the strength.

The paper is structured as follows. First, the model is briefly explained and the mechanics of single and double polymer chains with pre-defined cross-link topologies are discussed. Then, the mechanical performance of aligned fiber bundles containing three-fold coordinated cross-links is comprehensively investigated and compared to the case of two-fold coordination only. The influence of cross-link density and grafting density is discussed. Finally, the results are summarized, and possible future research directions are discussed.

## 2. Method

The inspiration of this work stems from metal-coordination bonds, where several (organic) ligands bind to a single metal cation [31]. Due to the long-range nature of the Coulomb force, a first principles treatment of such bonds is complicated [41,42] and a mechanical assessment of these bonds is even more scarce [20]. Therefore, we decided to choose a generic, coarse-grained approach to describe such bonds. While the used approach is tractable and simple enough to study rather large systems, it gives the possibility to explicitly study the influence of the coordination of cross-links on the mechanical behavior of polymeric systems which is the main objective of this work. As the model was already described elsewhere [37], here we only shortly recapitulate its main features and describe the additions necessary to model chain bundles.

**Figure 1:**
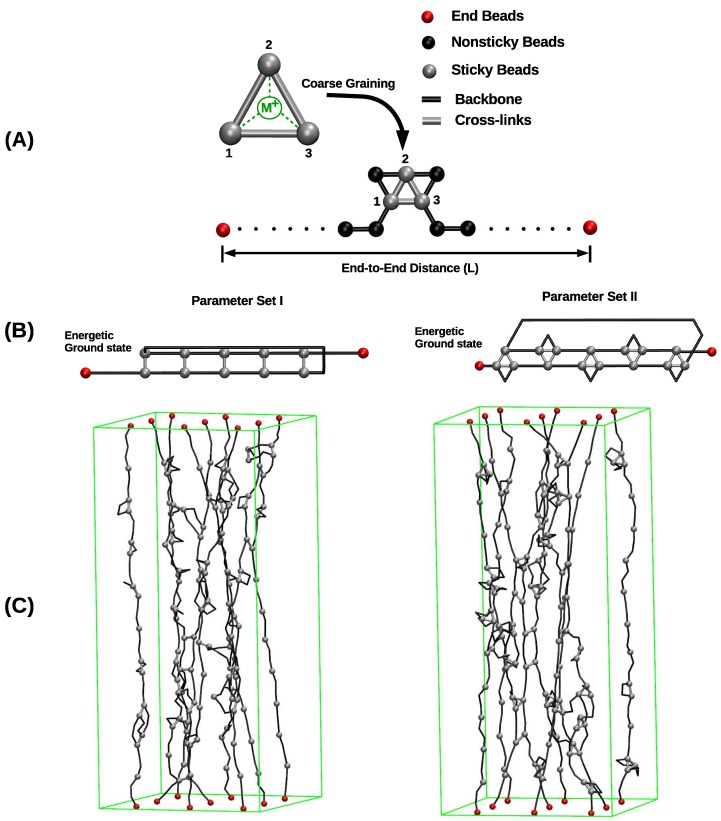
Overview of the investigated systems. (**A**) A linear polymer is described as a collection of covalently bound hard spheres, (black lines indicate the backbone of the polymer). Nonsticky, end, and sticky beads are shown in black, red, and gray, respectively. Sticky sites can form cross-links via coordination with metal ions. Cross-links are described in a coarse-grained manner via effective interactions between the sticky sites themselves (gray bars). Consequently, the metal ion and its interactions (dashed green lines) are not modeled explicitly. (**B**) The energetic ground state of cross-links in parameter sets I and II. Each cross-link complex (two-fold in parameter set I and three-fold in parameter set II) carries the same binding energy. (**C**) Snapshots from simulating a bundle of nine chains for parameter sets I (left) and II (right), respectively. In parameter set I, only two-fold coordinated cross-links may form. In parameter set II, three-fold coordinated cross-links are energetically most stable, but also two-fold coordinated cross-links have a nonzero binding energy. In the snapshot, gray bars and gray triangles correspond to two- and three-fold coordinated cross-links, respectively. It is clearly visible that no triangles are present in the system simulated with parameter set I. In the image nonsticky beads are removed for better visualization, end and sticky beads are shown. The green rectangular box represents the system size.

The systems investigated in this paper are either composed of one, two or nine polymer chains. $N_{C}$ denotes the number of beads per chain. In this study $N_{C}=50$ was chosen because typical protein segments containing metal-coordination bonds are relatively short consisting of only 30 to 80 amino acids [43]. The contour length of the polymer is given by $L_{c}=\left(N_{C}-1\right)r_{0}$, where $r_{0}$ is the equilibrium bond length. The first and last beads of each chain are permanently grafted to a substrate and are held fixed during the simulations. For the system consisting of nine chains, the grafting points are located on a square lattice with lattice constant *d* defining the grafting density. In the directions perpendicular to the chain axis, periodic boundary conditions are used (Figure 1).

The polymers are modeled using a bead-spring model of covalently bound hard spheres with radius *R*. The covalent interactions along the backbone are described using a Morse potential. The excluded volume and covalent interactions for two beads *i* and *j* (i≠j) can be written as follows:

(1) $$\begin{array}{ccc} U_{EV}\left(r_{ij}\right) & = & \left\{\begin{array}{cc} 0 & \mathrm{if}\;r_{ij}>R,\\ \infty & \mathrm{otherwise}. \end{array}\right. \end{array}$$

(2) $$\begin{array}{ccc} U_{Cov}\left(r_{ij}\right) & = & \left\{\begin{array}{cc} E_{0}\left[{\left(1-e^{-\alpha (r_{ij}-r_{0})}\right)}^{2}-1\right] & \mathrm{if}\;i,j\;\mathrm{covalently}\mathrm{linked},\\ 0 & \mathrm{otherwise}. \end{array}\right. \end{array}$$

The cross-links are introduced by declaring $N_{s}$ of the beads as “sticky”. Sticky beads refer to a possible ligand of a metal ion, thus only these sites can form cross-links. $\rho_{s}=N_{s}/N_{T}$ is the cross-link number density in the system, where $N_{T}$ denotes the total number of particles. The preferred coordination of cross-links is controlled using the framework provided by the REBO potential [38,39]. The main feature of REBO-like potentials is that the strength of a bond is dependent on the local coordination (i.e., the number of neighbors of a given particle). The relevant equations read as follows:

(3) $$U_{RCL}=\frac{1}{2}\sum_{<i\neq j>}\left[f_{R}\left(r_{ij}\right)+b_{ij}f_{A}\left(r_{ij}\right)\right]f_{c}\left(r_{ij}\right),$$

(4) $$f_{R}=Ae^{-\lambda r_{ij}},\;f_{A}=-Be^{-\mu r_{ij}},$$

The sum runs over all pairs of sticky sites. $f_{R}$ and $f_{A}$ are pair-wise repulsive and attractive contributions, respectively. $f_{c}\left(r_{ij}\right)$ is a continuous cut-off function:

(5) $$f_{c}\left(r_{ij}\right)=\left\{\begin{array}{cc} 1 & \mathrm{if}\;r_{ij}<R_{0},\\ \frac{1}{2}\left(1+\cos\left[\pi \frac{r_{ij}-R_{0}}{\Delta }\right]\right) & \mathrm{if}\;R_{0}<r_{ij}<R_{0}+\Delta ,\\ 0 & \mathrm{if}\;r_{ij}>R_{0}+\Delta , \end{array}\right.$$

The bond-order parameter $b_{ij}$ depends on the local environment of each sticky site (i.e., the bond angle $\theta_{ijk}$ between three sites):

(6) $$\begin{array}{ccc} b_{ij} & = & {\left(1+\beta^{n}\xi^{n}\right)}^{-\frac{1}{2n}},\\ \xi & = & \sum_{k\neq i,j}g\left(\theta_{ijk}\right)f_{c}\left(r_{ik}\right),\\ g(\theta_{ijk}) & = & 1+\frac{c^{2}}{d^{2}}-\frac{c^{2}}{d^{2}+{\left(h-\cos\theta_{ijk}\right)}^{2}}. \end{array}$$

The sum in ξ runs over all sticky sites *k* (except for *i* and *j*) inside the cut-off region of site *i*. In particular, REBO potentials are reactive, meaning that bond formation, rupture, and reformation are explicitly taken into account without any external input. The values of the parameters used in the simulations are summarized in Table 1.

In the chosen approach, metal-coordination complexes are described in a coarse-grained manner via effective interactions between the ligands (the sticky sites). In this description, the metal ion itself is not explicitly considered (see Figure 1A). It would be desirable to obtain the effective interaction parameters needed for the REBO potential via detailed ab initio calculations of the coordination complexes. Unfortunately, such calculations are difficult and only a limited number of investigations on the mechanics of metal-coordination complexes using first principles approaches exist [20,44,45]. Thus, the parameters used in this work were chosen empirically to fulfill three constraints. First, the binding energy of a cross-link complex is approximately a quarter of a covalent bond. Second, a tris-complex is energetically the most stable complex in the system. Third, the equilibrium bond length of the cross-links and covalent bonds are identical. It is evident that this assumption is only an approximation to the real situation, because the exact value of the cross-link bond length will depend on the size of the ligand and the metal ion. Nevertheless, the approximation is justified as in the coarse-grained description one bead roughly corresponds to one amino acid that is much larger than the spatial extension of the metal-coordination bond, whereas in the coarse-grained description the cross-link bond length is the distance of the center of masses of the amino acids. The numerical values of the parameters fulfilling these constraints and used in the simulations are summarized in Table 1.

The focus of this work is to elucidate the influence of the coordination of cross-links on the mechanical behavior of polymeric systems. This is done by comparing the mechanical behavior of two systems having the same number and distribution of sticky sites but differing in the coordination of the formed cross-links. In the first system, a two-fold coordinated dimer is the only stable cross-link that may form (parameter set I). In the second system, the energetically most stable form of cross-links is a three-fold coordinated trimer, but also a two-fold coordinated dimer has a nonzero binding energy (parameter set II). Figure 1B schematically shows the topology of cross-links in their energetic ground state for the two parameter sets. The two parameter sets used for modeling the cross-links in these two systems are listed in Table 1. The parameters are chosen such that the energy of one complex as well as the equilibrium bond length are the same in both systems but note that the binding energy of two-fold coordinated complexes is different in the two parameter sets. In the current work, displacement controlled uniaxial computational loading tests have been performed. Using Monte Carlo (MC) simulations, the polymer was stretched gradually from an initial compressed state characterized by a small end-to-end distance *L* until the system failed via rupture of all chains above the contour length $L_{c}$. The elongation is performed by fixing the first beads of all chains at z=0 and the last beads of all chains at the desired reduced elongation $L/L_{c}$. For each elongation, the load is computed by averaging 5000 independent configurations obtained by a standard Metropolis algorithm [46]. Then, the elongation is increased by $\Delta L/L_{c}=0.0002$ for all polymer chains simultaneously. This loading geometry is chosen to mimic the case where heavily cross-linked parts of one protein are sandwiched between considerably stiffer collagen-like domains as found, e.g., in the mussel byssus [7].

## 3. Results and Discussion

### 3.1. Single Chain

**Figure 2:**
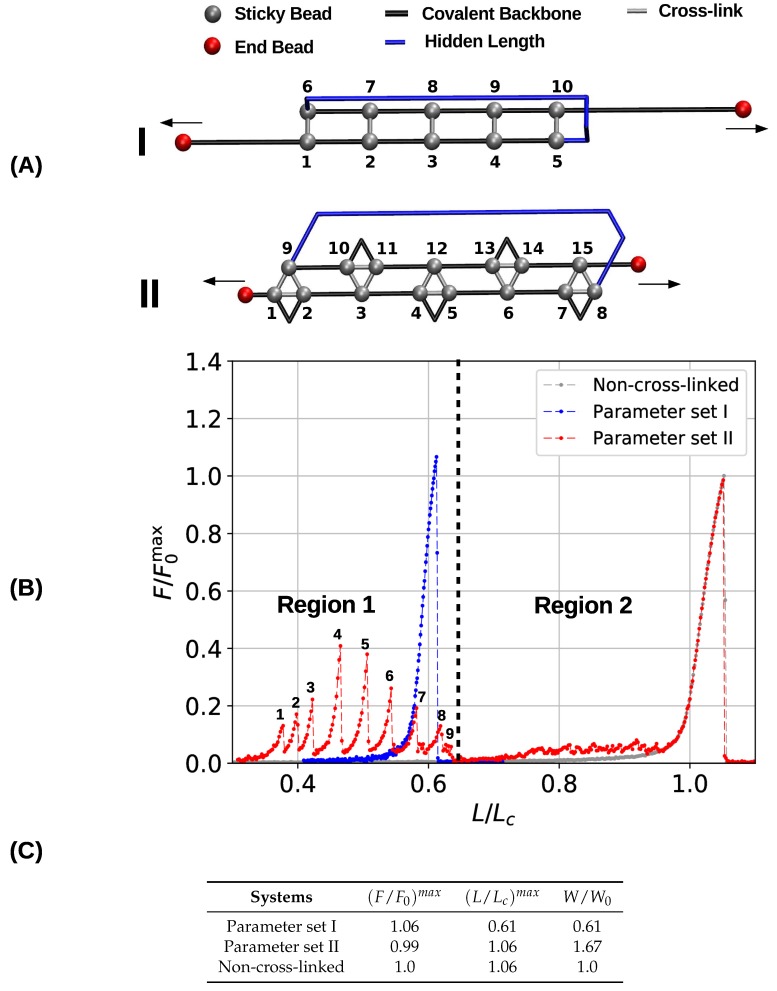
Initial configurations (**A**) and corresponding load-displacement curves (**B**) of systems containing five cross-link complexes of different coordination. The topologies are designed such that they resemble a β-sheet structure and that the cross-links are loaded in parallel. The chains have a total number of 50 monomers. In the load-displacement curves, the blue and red lines correspond to the two- (parameter set I) and three-fold coordinated system (parameter set II), respectively. The gray line denotes the deformation of a reference system without any cross-links. (**C**) Strength (${\left(F/F_{0}\right)}^{max}$), maximum extension (${\left(L/L_{c}\right)}^{max}$) and work-to-fracture ($W/W_{0}$) of the different systems. Strength is normalized with the strength of a non-cross-linked system, and work-to-fracture is normalized with the work-to-fracture of the non-cross-linked system.

To gain a first understanding of the influence of the topology of cross-links on the mechanical performance of cross-linked fibers, first a single chain with a pre-defined topology of cross-links was studied. Figure 2A shows the initial topology of the investigated two- and three-fold coordinated structures. Here, the sticky sites are numbered consecutively, and cross-links are indicated by gray bars, black lines depict the covalent backbone. Following the definition given elsewhere [4], we refer to the hidden length (indicated by the blue color) as the part of the backbone that is shielded from loading by the cross-links. In both systems (parameter sets I and II) the cross-link complexes carry the same amount of energy by construction. This means that although the systems differ in sticky-site density, their ground state energy is the same. The topology of cross-links is chosen such that the complexes are loaded in parallel leading to an effective shear force on the backbone. Such a topology distributes the applied load over all cross-links, meaning that the strength of the system is correlated with the number of cross-links [29,47]. This cross-link topology resembles a minimal (parallel) β-sheet structure and can be found in many mechanically strong biological materials such as spider silk, amyloid fibers and muscle protein titin [15,23,48]. Corresponding load-displacement curves are shown in Figure 2B. The relative force (i.e., the force relative to the strength of the non-cross-linked polymer $F/F_{0}^{max}$) is plotted as a function of the reduced length (i.e., the end-to-end distance over the contour length $L/L_{c}$). Ultimate strength is defined as the maximum load a structure can take. In particular, the strength is the maximum load observed in the force-extension curve.

The obtained load–displacement curves show that in the case of parameter set I five cross-links forming the shown topology are sufficient to rupture the backbone of the polymer before the contour length is reached. In the load–displacement curve (blue line in Figure 2B) no cross-link rupture can be observed; the rise of the load around an elongation of approximately 0.6 corresponds to backbone rupture. In the chosen β-sheet-like topology the cross-links are loaded in parallel, meaning that they cooperatively deform and share the load. Because the energy (and strength) of one two-fold complex is equal to the quarter of one covalent bond, five of these complexes are sufficient to take more load than the backbone. Such a premature rupture of the backbone will not be observed when less than five cross-link complexes are present. In this case, the sacrificial bonds rupture one by one leaving the backbone intact [40]. Furthermore, the strength of the system (parameter set I) is slightly higher than the strength of the non-cross-linked polymer ${\left(F/F_{0}\right)}^{max}=1.06$. This slight increase stems from the fact that the system fails at extensions below one. This effectively hinders large fluctuations of the backbone that are known to deteriorate the mechanical behavior [49,50].

**Figure 3:**
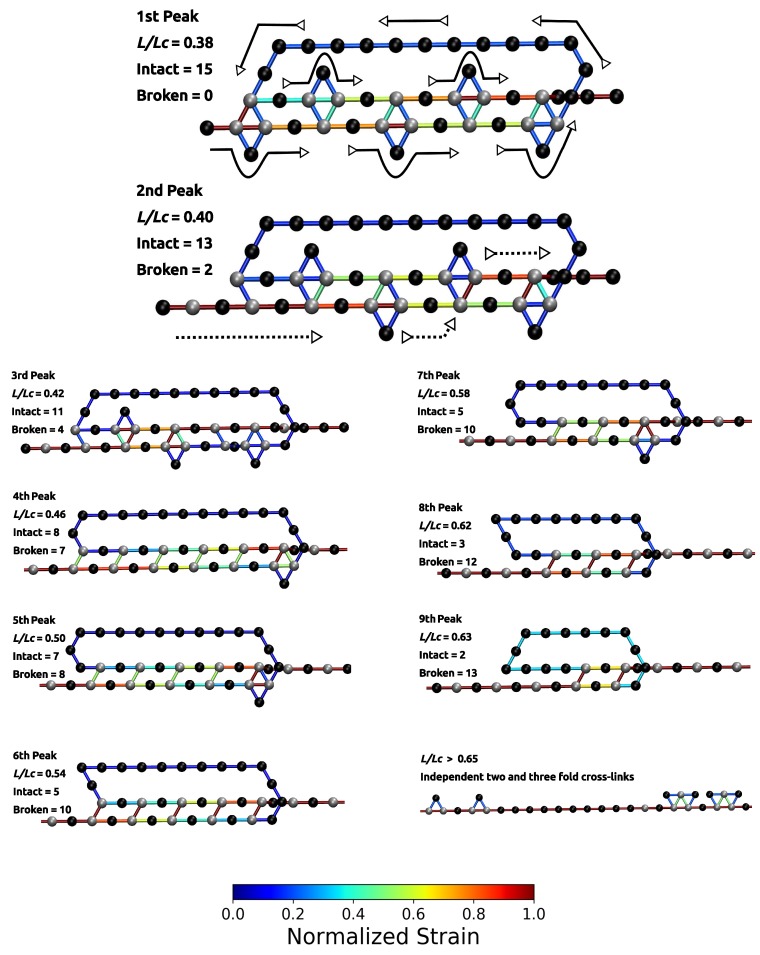
Polymer topology and strain distribution in each bond close to bond rupture for parameter set II. Strain in cross-links and in covalent bonds is normalized with corresponding maximum strains. The numbering of peaks corresponds to the numbering given in Figure 2. The first two images are enlarged to better visualize the course of the backbone (solid arrows) and the flow of the strain (dashed arrows). It is evident that the investigated cross-link topology shields a large portion of the backbone from loading (hidden length).

The load–displacement curve of the system simulated with parameter set II shows a more complex mechanical behavior than the classical two-fold coordinated system (red curve in Figure 2B). Although the binding energy of a single cross-link complex is the same in both systems, no premature rupture of the covalent backbone (i.e., failure of the backbone before the contour length) is observed in the three-fold coordinated system. Comparison with the non-cross-linked reference system shows that the system recovers its extensibility, while maintaining its strength. Furthermore, the system shows a dramatic increase in the work-to-fracture *W* compared to the other two systems. The work to fracture is a measure of the toughness of the system and can be obtained by calculating the area under the load–displacement curve. The work-to-fracture is normalized with the work-to-fracture of the non-cross-linked reference system $W_{0}$. It is found that $W/W_{0}$ increases from 0.61 to 1.67, from the two-fold coordinated system (parameter set I) to the three-fold coordinated system (parameter set II). The load–displacement curve obtained for parameter set II can be roughly separated in two distinct regions: region 1 ranges approximately from $L/L_{c}=0.3$ to 0.7 and consists of several peaks of different height; region 2 starts at extensions larger than 0.7 and shows a much more continuous loading, where no single peaks can be observed (see Figure 2B). Each force peak observed in region 1 corresponds to the (partial) rupture of one cross-link complex. Figure 3 shows the mean strain in the structure at peak load (the numbering of the peaks corresponds to the numbering given in the load–displacement curve in Figure 2B). The graphs corresponding to the first two peaks are enlarged to better visualize the course of the backbone (solid arrows in the graph corresponding to peak 1) and the flow of the strain through the structure (dashed arrows in the graph corresponding to peak 2). For each snapshot also the corresponding extension $L/L_{c}$ and the number of intact and broken bonds are given. Comparison of the backbone and of the force flow shows that the investigated topology shields a large portion of the backbone from being loaded (the hidden length). Following the subsequent rupture events, it can be seen how the three-fold coordinated complexes gradually decompose into two-fold coordinated complexes. It needs five rupture events until this process is completed and the initially three-fold coordinated complexes are completely transformed into two-fold coordinated complexes. Then, the two-fold coordinated cross-links rupture one by one until the hidden length is completely unraveled (at $L/L_{c}\approx 0.65$). At this point, all cross-links are open. Nevertheless, because the bonds are reversible the unfolding of the hidden length gives the possibility to reformation of some bonds. Most of the reformed cross-links are of independent topology [49]. The slight increase in load in the second region of the load–displacement curve compared to the non-cross-linked case corresponds to the rupture of these newly formed independent cross-links.

The load-displacement curve as well as the corresponding mechanical parameters (Figure 2B,C) show clearly that the deformation behavior of three- and two-fold coordinated cross-link complexes is very different, although both carry the same amount of binding energy. From a topological perspective, this difference stems from a different geometric arrangement of bonds. While the two-fold coordinated complexes may form a topology that is loaded in pure shear via a cooperative deformation of all bonds, three-fold complexes consist of three bonds, two of which loaded cooperatively, and one loaded in direction of the backbone. As an example, consider the first three-fold complex consisting of sticky beads 1, 2 and 9 in Figure 2A. Effective bonds 1–9 and 2–9 are loaded cooperatively, while bond 1–2 is loaded along the backbone. The same observation holds true for each three-fold coordinated complex—out of three effective bonds, two are loaded in parallel, and one along the backbone. It is these bonds along the backbone that are loaded noncooperatively and rupture first, thus transforming the three- into two-fold coordinated complexes. These bonds rupture independently, thus preventing the structure from backbone failure.

### 3.2. Two Chain System

**Figure 4:**
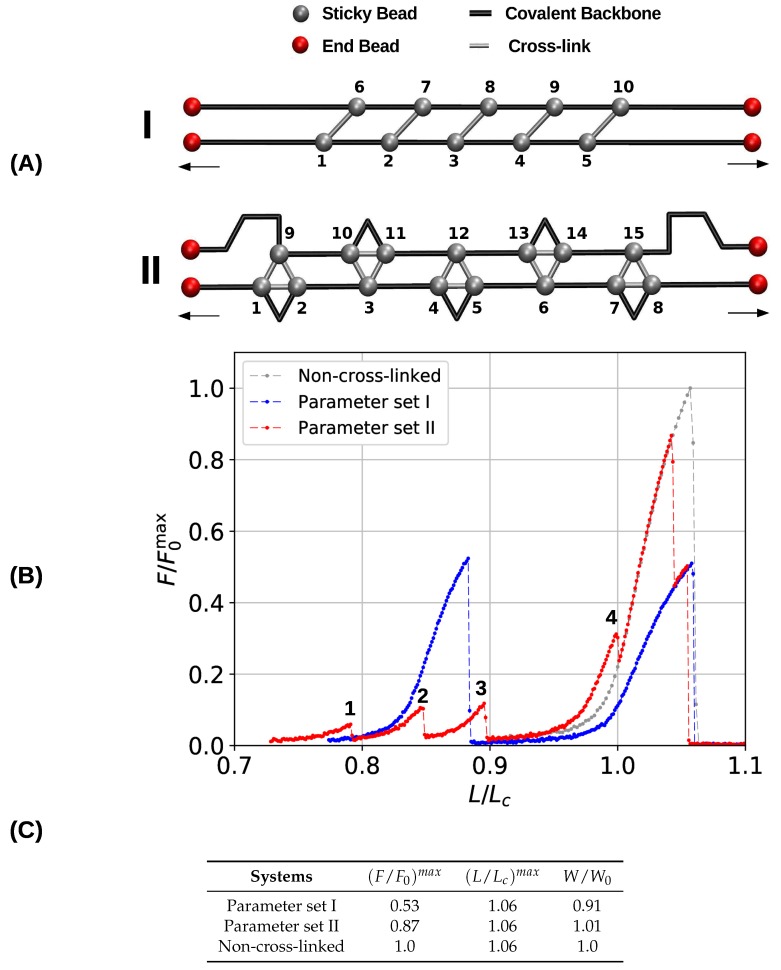
(**A**) Starting topology and (**B**) load–displacement curves of systems consisting of two chains and simulated with parameter sets I and II, respectively. Both systems show a similar extensibility as the non-cross-linked system, while the strength of the three-fold coordinated system (parameter set II) and the reference system is significantly higher compared to the system forming two-fold coordinated cross-links only (parameter set I). (**C**) Corresponding strength (${\left(F/F_{0}\right)}^{max}$), maximum extension (${\left(L/L_{c}\right)}^{max}$), and work-to-fracture ($W/W_{0}$).

In a next step, we investigated the behavior of a system consisting of two parallel linear chains. In contrast to a single chain, a multichain system allows also for the presence of interchain cross-links, which have been shown to play a decisive role in the mechanics of aligned chain bundles [40]. Figure 4A shows the investigated topology of two- and three-fold coordinated complexes. This topology is similar to the one investigated for the single chain (Figure 2) but is formed between two chains instead of only one. Each chain has a length of 23 beads and contains five cross-link complexes. The topology is designed such that most of the cross-links are interchain (the two-fold coordinated case consists solely of interchain cross-links). The lateral distance between the chains was set equal to one covalent bond length. Corresponding load–displacement curves are shown in Figure 4B. Blue and red lines represent the mechanical response of the systems simulated with parameter sets I and II, respectively, and the gray line corresponds to the bare non-cross-linked polymer as reference.

Figure 4B shows the obtained load–displacement curves by plotting the relative force $F/F_{0}^{max}$ versus the reduced length $L/L_{0}$ (as before, $F_{0}^{max}$ is the strength of the non-cross-linked system). The system containing two-fold coordinated cross-links only (blue line) shows the same behavior as was already reported elsewhere [40]. In the non-cross-linked case, both chains are elongated simultaneously until they rupture when the chains are elongated above their maximum strength. Because the chains are identical also their strength and maximum displacement are identical, meaning that their strengths add up. Furthermore, the chains rupture simultaneously. This behavior changes in the cross-linked case. As the topology of cross-links is chosen such that the cross-links are loaded cooperatively each cross-link may be loaded up to its strength which is approximately a quarter the strength of the backbone. Consequently, five cross-links in parallel can take up more load than the backbone of the structure. This leads to the failure of the backbone of one of the chains and releases the load from the cross-links (peak at $L/L_{c}\approx 0.88$). The remaining intact backbone then fails at its maximum extension $L/L_{c}\approx 1.06$. Because the two chains do not fail simultaneously, the strengths of the two chains do not add up and, consequently, the strength of the system is reduced approximately by a factor of two compared to the non-cross-linked system, where both chains take the load cooperatively. Thus, the presence of cross-links effectively weakens the system. Nevertheless, this effect can be partially reversed, when some disorder is introduced in the system. Cooperative loading of the cross-links is possible only, if the cross-links are spaced regularly [51]. Whenever some disorder is introduced in their spatial distribution, the load experienced by the cross-links at a given extension will be different and they will fail sequentially not exceeding the strength of the backbone.

The mechanical behavior of the three-fold coordinated system is different (red line in Figure 4B). The topology is designed such that most of the cross-links form (effective) interchain bonds. Nevertheless, having two chains only, it is not possible to prevent the formation of intrachain cross-links when all possible bonds should be closed. The main difference in the mechanical behavior compared to the system simulated with parameter set I is that the polymer backbone does not rupture prior to the contour length. This improves the mechanical properties such as strength and work-to-fracture of the system containing three-fold coordinated cross-link compared to the dimer only case. Values for the mechanical parameters are shown in Figure 4C. The strength ${\left(F/F_{0}\right)}^{max}$ increases from 0.53 to 0.87. While, the presence of cross-links weakens a multichain system in general due to presence of interchain cross-links that are loaded cooperatively, this weakening is strongly reduced for the three-fold compared to the two-fold coordinated system. In contrast to the single chain system discussed in the previous section, the work-to-fracture scales only slightly with the coordination, increasing roughly only 10% from 0.91 to 1.01.

**Figure 5:**
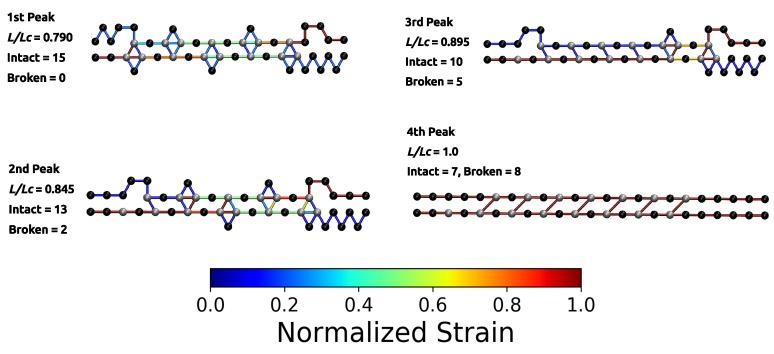
Topology and strain distribution for the system consisting of two chains. Strain in cross-links and in covalent bonds is normalized with corresponding maximum strains. The numbering of peaks corresponds to the numbering given in Figure 4. As in the single chain case the deformation behavior is such that three-fold coordinated complexes first transform into two-fold coordinated ones, until the system is purely two-fold coordinated at peak 4.

While the load–displacement curve of the system simulated with parameter set I consists of two large peaks that correspond to the backbone rupture of the two involved chains, the corresponding curve of the system containing three-fold coordinated cross-links is more structured. Before the structure finally fails, several smaller peaks (labeled from 1 to 4 in Figure 4) can be observed. These correspond to the rupture and reformation of cross-links. Figure 5 shows the strain distribution in each bond before the rupture event directly at peak load. The load is distributed such that the intrachain cross-links rupture first. This leads to the formation of a purely two-fold coordinated topology consisting solely of interchain cross-links. The transformation of three-fold into two-fold topology is completed when peak 4 is reached. In total, seven two-fold coordinated complexes have formed and peak 4 corresponds to their rupture. Exactly at $L/L_{c}=1$, the load is shifted to the backbone. From now on covalent bonds start elongating until the structure fails.

### 3.3. Aligned Fiber Bundle

After having discussed the simplified situations of one and two chains, now the more realistic situation of an aligned fiber bundle consisting of nine chains shall be considered. The starting configurations are prepared by slowly reducing the end-to-end distance of a system without sticky sites from $L/L_{c}=1$ to 0.15. Then, $N_{s}$ randomly chosen beads are declared sticky, while it is ensured that two sticky beads are not direct neighbors along the chain. The system is equilibrated, and cross-links may form between sticky sites that are spatially close. To obtain load–displacement curves, the system is stretched until all chains are ruptured at elongations of $L/L_{c}=1.1$. The presented curves are averages over 48 independent starting configurations. Strength, stiffness, work-to-fracture and the elongation at which the first chain rupture occurs are computed as a function of sticky-site density ($\rho_{s}=N_{s}/N_{T}$) and grafting density (defined by the lateral distance *d* between the chains). The grafting density is strongly influencing the ratio of inter- to intrachain cross-links and is, thus, one main factor determining the mechanical behavior of flexible, aligned fiber bundles. It has been shown that the interchain cross-links are responsible for a dramatic decrease in the strength of two-fold coordinated systems [40].

**Figure 6:**
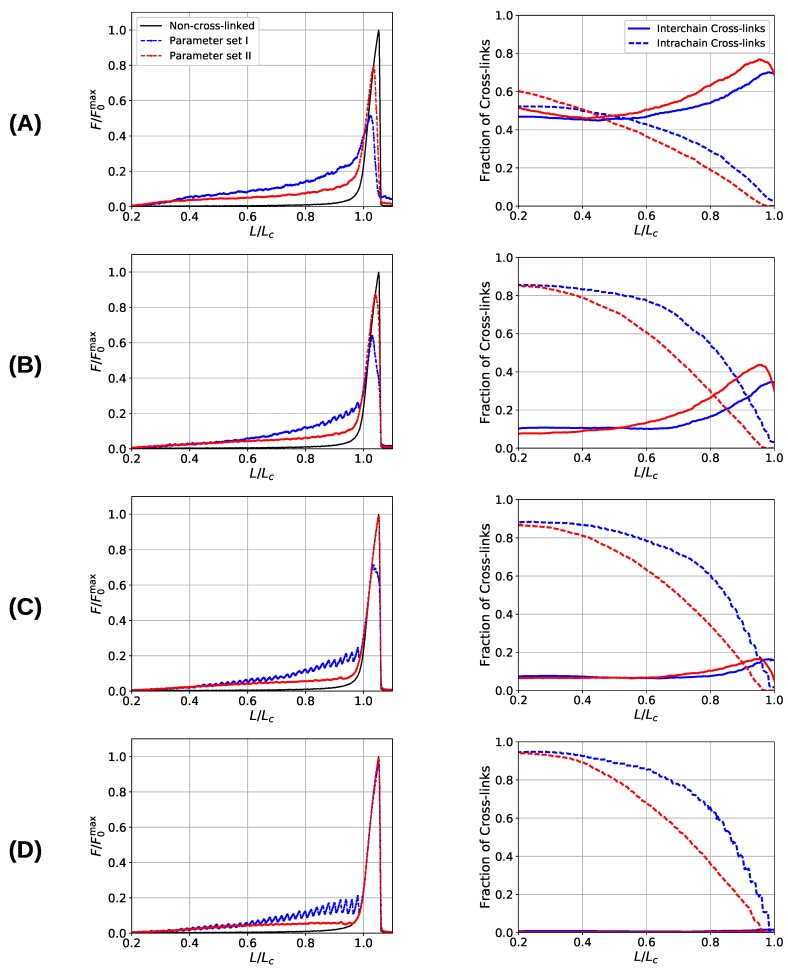
Load–displacement curves (**left**) and fraction of inter- and intrachain cross-links (**right**) of a system consisting of nine aligned chains. Different rows correspond to different grafting densities, defined by the lateral distance d/R of the grafting points of the chains: d/R= (**A**) 7.5, (**B**) 15, (**C**) 22.5, and (**D**) 30. Blue and red lines correspond to systems simulated with parameter sets I and II, respectively. The black line indicates the response of the non-cross-linked reference curve. The sticky-site density was $\rho_{s}=0.36$ for all curves. Data are shown as the average over 48 independent simulation runs.

Figure 6 shows the load–displacement curves of the two investigated systems as well as the number of inter- and intrachain cross-links for several grafting densities (d/R=7.5, 15.0, 22.5, and 30), respectively. The load–displacement curves show that the system containing three-fold coordinated cross-links shows in general a higher strength compared to the system containing solely dimers. Only in cases where the chains are that much separated that no interchain cross-links may form and the chains deform completely independently (separations larger than d/R=30.0) the strength of both systems coincides, approaching the strength of the non-cross-linked polymer. Furthermore, the load–displacement curve of the three-fold coordinated system shows a much smoother course than the corresponding load–displacement curve of the two-fold coordinated system. In particular, the rupture of single peaks is clearly visible in the latter system, especially in the two cases of lowest grafting density (i.e., d/R=22.5 and 30).

**Figure 7:**
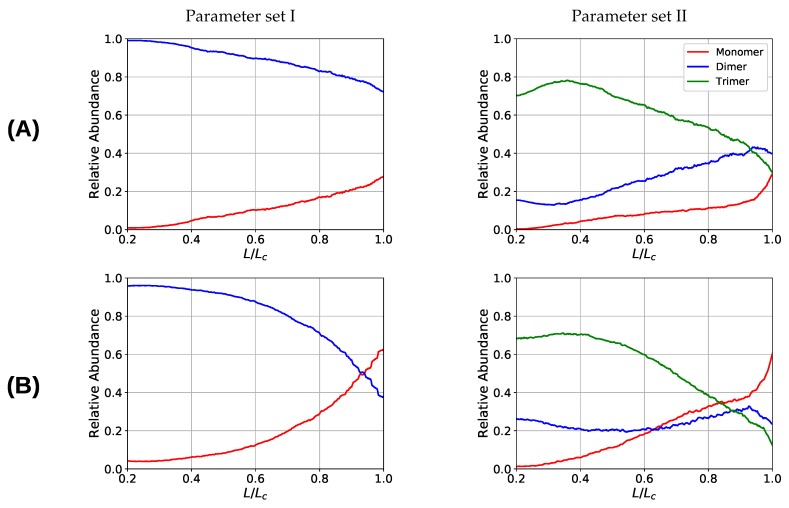

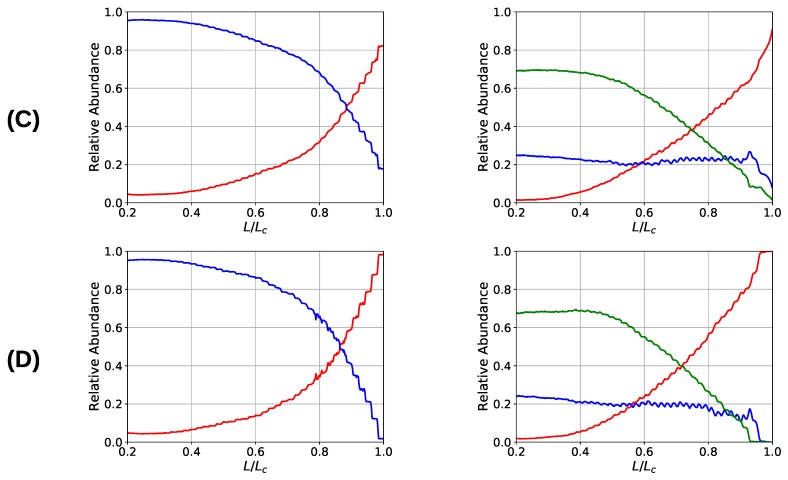
Coordination plot of the systems simulated with parameter set I (**left**) and II (**right**) for all investigated grafting densities: d/R= (**A**) 7.5, (**B**) 15, (**C**) 22.5, and (**D**) 30; showing the fraction of cross-links of different coordination in the system. For parameter set II at the onset of deformation most cross-links are three-fold coordinated, but also a considerable number of dimers is present that possess a nonzero binding energy. Important observations are, first, that the number of intact cross-links at contour length increase with increasing grafting density and, second, that for parameter set I the number of cross-links is strictly decaying with elongation, while for parameter set II and high grafting densities (**A**) and (**B**) the number of dimers increases on cost of the number of trimers.

One observation in the microscopic deformation behavior of the two investigated systems is that the number of intrachain cross-links decays much faster in the three-fold coordinated compared to the two-fold coordinated system (see Figure 6, right column). This is because in the system containing trimers, it is the trimers that rupture first as can be seen in Figure 7. The coordination plot shows the relative abundance of cross-links of different coordination as a function of elongation for the four investigated grafting densities (Figure 7A–D). At the lowest grafting density d/R=30.0 (Figure 7D), the behavior of intrachain cross-links can be directly visualized because due to the large distance of the chains no interchain cross-links are present. Figure 7D shows that starting from $L/L_{c}\approx 0.4$ the number of trimers drastically decreases. The number of dimers shows a much less pronounced decrease over the entire deformation range. A sharp decrease to zero can only be seen shortly before the contour length is reached. This confirms that in systems containing three-fold coordinated cross-links it is mostly the rupture of trimers that is responsible for the decline in intrachain cross-links. While a broken trimer removes two bonds out of the system, in the two-fold coordinated system a rupture of a dimer reduces the number of bonds only by one. It is this disproportion that is responsible for the faster decline of intrachain cross-links in the system containing trimers compared to the purely two-fold coordinated one. In parameter set II, it is surprising that not all cross-links are three-fold coordinated from the onset of deformation, but a considerable number of dimers is present. This is because the formation of a three-fold coordinated cross-link proceeds by attachment of a monomer to an already formed dimer. When no sticky monomers are left because they are all bound to a dimer state, the system is kinetically stabilized, because for the formation of an—energetically more stable—trimer, first a dimer must dissolve to provide free monomers. Another important observation is that with increasing grafting density the number of intact cross-links at the contour length $L/L_{c}=1$ also increase. This can be solely attributed to interchain cross-links because no intrachain cross-links can persist at this elongation. Furthermore, for the simulations with parameter set I the number of cross-links decreases with increasing elongation. This is different for the system described with parameter set II and high grafting densities (Figure 7A,B). While the number of trimers decays, the number of dimers increases steadily until a small decrease can be observed shortly before reaching the contour length. Interestingly, for the same grafting density the number of monomers (sticky sites that are not part of any cross-link) reaches approximately the same amount for both systems.

**Figure 8:**
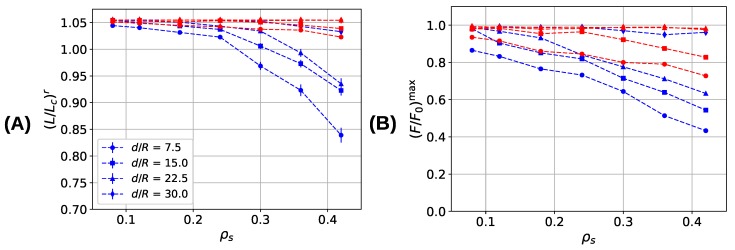

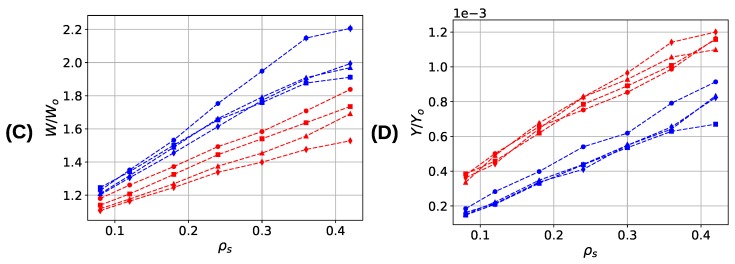
Mechanical properties of cross-linked fiber bundles for different sticky-site ($\rho_{s}$) and grafting densities. Blue and red lines correspond to systems simulated with parameter sets I and II, respectively. (**A**) Appearance of the first backbone rupture ${\left(L/L_{c}\right)}^{r}$, (**B**) strength ${\left(F/F_{0}\right)}^{max}$, (**C**) work-to-fracture $W/W_{0}$, and (**D**) stiffness $Y/Y_{0}$.

Figure 8 summarizes the mechanical properties of the investigated systems as a function of sticky-site density and grafting density. Shown are the extension at which the first backbone rupture occurs ${\left(L/L_{c}\right)}^{r}$ (Figure 8A), the strength ${\left(F/F_{0}\right)}^{max}$ (Figure 8B), the work-to-fracture $W/W_{0}$ (Figure 8C), and the stiffness $Y/Y_{0}$ (Figure 8D). Blue and red lines correspond to the properties of the systems simulated with parameter sets I and II, respectively. The grafting density can be used to tailor the behavior of the system from noninteracting independent chains (d/R=30) with zero interchain cross-links to highly interacting chains characterized by many interchain cross-links. An important observation is that in the three-fold coordinated system the first polymer backbone rupture occurs above the contour length for all investigated sticky-site and grafting densities (i.e., ${\left(L/L_{c}\right)}^{r}>1.0$). This behavior is different to the two-fold coordinated system where ${\left(L/L_{c}\right)}^{r}<1.0$ for interacting chains and high sticky-site densities. It is this premature rupture of the backbone that is responsible for the dramatic loss in strength for the two-fold coordinated system at high sticky-site densities as can be seen in Figure 8B. For high cross-link densities, the strength in the system simulated with parameter set I decreases almost by a factor of two compared to the noninteracting case. Also, in systems simulated with parameter set II a decline in the strength can be observed for high cross-link densities, but this loss in strength is much less pronounced than in the former system. The system containing three-fold coordinated cross-links possesses a higher strength than the dimer system for all grafting densities except for d/R=30, where the strength becomes independent of sticky-site density and approaches the strength of a non-cross-linked polymer. The average number of nonruptured chains at contour length is a direct measure of the strength. As this number is higher in the three-fold coordinated system, this system shows a higher strength than the two-fold coordinated system. Responsible for the premature rupture of the backbone are interchain cross-links that increase in number with increasing sticky-site density and increasing grafting density (see Figure 6). Interchain cross-links may effectively transmit forces from one chain to the other. Many of such cross-links may be loaded cooperatively leading to an effective shear force on the backbone exceeding the strength of the backbone (see also Figure 4).

The work-to-fracture is the energy needed to elongate the system until the system permanently fails (Figure 8C). The work-to-fracture heavily depends on the number of cross-links present in the system that need to be broken before the system fails. Thus, it is not surprising that the work to fracture increases with increasing sticky-site density and increasing grafting density in both systems. More surprising is that the two-fold coordinated system consistently shows a higher work to fracture than the three-fold coordinated system for all studied sticky-site and grafting densities although the energy of each complex is the same. This is because, first, at the onset of deformation only roughly 70% of the cross-links are three-fold coordinated (see Figure 7). The remaining 30% have a lower coordination and, thus, a lower binding energy. Second, although the sticky-site density is the same in both systems, the number of cross-link complexes can differ considerably. A sticky-site density of 24% allows for the formation of 54 bis-complexes, but only 36 trimers. Consequently, at the same sticky-site density the ground state energy of the system simulated with parameter set I is lower than the one for the system described with parameter set II.

The stiffness of a material is defined as its resistance against deformation and it is measured as the initial slope of the load–displacement curve. Figure 6 shows that cross-linked systems show in general a higher resistance against deformation and thus, possess a higher stiffness $Y/Y_{0}$. This observation is confirmed by the numerical values of the stiffness as depicted in Figure 8D. The stiffness is normalized with the stiffness of a single cross-link bond $Y_{0}$ obtained as the second derivative of the used potential (parameter sets I and II) evaluated at equilibrium bond length. The results indicate that the stiffness shows a strong dependence on the number of cross-links present, while the grafting density is only of little influence. Furthermore, the system containing trimers (parameter set II) shows a consistently higher stiffness than the system containing dimers only (parameter set I). This is because the number of bonds is higher in the first compared to the latter system. Although—for the same sticky-site density—the maximum number of dimers is larger than the maximum number of trimers, the number of bonds is higher in the three-fold coordinated system. Analysis of the microscopic details shows that for the investigated structures the ratio of the number of bonds is relatively constant ranging from 1.5 to 2, a value that is close to the ratio of observed stiffnesses. This result indicates that the differences in stiffness are caused by the different number of bonds in the two investigated systems.

## 4. Conclusions

The influence of the coordination of cross-links on the mechanical response of aligned fiber bundles was investigated. In particular, two systems differing in their coordination of cross-links were compared. In the first system, a classical cross-link of two-fold coordination is the only cross-link that may form, while in the second system a three-fold coordinated cross-link is the most stable configuration. The energetics of the cross-links were chosen such that both cross-link complexes (the dimer as well as the trimer) carry the same binding energy. To analyze the effect of coordination, simple systems consisting of one and two chains were studied, prior to the investigation of a more realistic bundle containing nine chains. The results clearly show that the coordination of cross-links is an important parameter to understand the mechanical performance of fiber bundles. The main finding is that a higher coordination of cross-links partially reverts the reduction in strength that was recently predicted for classical two-fold coordinated cross-links [40]. This is because three-fold coordinated systems effectively prevent premature rupture of the covalent backbone frequently observed in two-fold coordinated systems.

The work-to-fracture and stiffness have also been computed for the two systems. The two-fold coordinated systems shows a higher work-to-fracture, but a reduced stiffness compared to the three-fold coordinated one. The work-to-fracture mostly depends on the number of closed cross-link complexes in the system. Having the same sticky sites density, the possible number of cross-link complexes is higher in the first system compared to the latter. The main factor determining the stiffness is the number of bonds present, the grafting density is only of little influence.

Metal-coordination bonds often found as cross-linkers in biological materials are the source of inspiration for the presented work. The results suggest that living organisms may tune the mechanical properties of some of their load bearing entities by tailoring the coordination of cross-links and/or their density. The different mechanical behavior of inter- and intrachain cross-links indicate that the mechanical performance of fiber bundles can be most effectively enhanced by incorporating strong intrachain and weak interchain cross-links. This is because interchain cross-links are often loaded cooperatively causing the premature rupture of the backbone and, thus, degrading the overall strength of the bundle. Recently, it was shown experimentally that the mechanical properties of an elastomer could be greatly enhanced by incorporating weaker hydrogen bonds and stronger metal-coordination bonds into a chemically cross-linked network [52]. The effect of the presence of bonds of different strength will be investigated in future simulation studies. 

## Figures and Tables

**Table 1:** Simulation parameters describing covalent bonds and reversible cross-links.

Covalent Bonds		Cross-Links	Parameter Set I (Dimers only)	Parameter Set II (Trimers and Dimers)

$E_{0}/k_{B}T$	200.0	A$/k_{B}T$	1,101,324	367,108
*R*	-	B$/k_{B}T$	14,840	5,396
$r_{0}/R$	3.0	λ·R	3.33	3.33
α·R	2.0	μ·R	1.66	1.66
		*n*	0	4
		β	-	1.0
		$h=\cos\theta_{0}$	-	0.5
		*c*	-	8.0
		*d*	-	2.0
		$R_{0}/R$	4.2	4.2
		Δ/R	1.2	1.2

The unit of length is set by the hard sphere radius *R*, the energy scale is given by $k_{B}T$. The two parameter sets given correspond to a system that can only form dimers (I) and where the most stable cross-link is a trimer (II). The parameters are chosen such that the binding energy of one complex is a quarter of a covalent bond.
